# Characterization of isolated pigeon paramyxovirus-1 (PMV-1) and its pathogenicity in broiler chickens

**Published:** 2017-03-15

**Authors:** Mansour Mayahi, Masoud Reza Seyfi Abad Shapouri, Ramezan Ali Jafari, Mehrdad Khosravi Farsani

**Affiliations:** 1 *Department of Clinical Sciences, Faculty of Veterinary Medicine, Shahid Chamran University of Ahvaz, Ahvaz, Iran; *; 2 *Department of Pathobiology, Faculty of Veterinary Medicine, Shahid Chamran University of Ahvaz, Ahvaz, Iran; *; 3 *DVSc Graduate of Avian Health and Diseases, Department of Clinical Sciences, Faculty of Veterinary Medicine, Shahid Chamran University of Ahvaz, Ahvaz, Iran.*

**Keywords:** Broiler chicken, Isolation, Newcastle disease, Paramyxovirus-1, Pigeon

## Abstract

Characterization of isolated pigeon paramyxovirus-1 (PMV-1) and its pathogenicity in broiler chickens were studied. Two hundred and thirty-two samples collected from 50 unvaccinated pigeons lofts suspected to Newcastle disease from private houses and bird markets from Ahvaz, Iran. Swab samples from cloaca and oropharynx of live pigeons and from trachea, lung, liver, spleen, kidney, brain, proventriculus and cecal tonsil of dead pigeons suspected to ND were collected. Isolation of the PPMV-1 was performed through intra-allantoic inoculation of 9- to 11- day-old embryonated chicken eggs. The RNA extraction and cDNA synthesis were conducted. With PCR, multiplication of cleavage site of F gene was carreid out and PCR products were sequenced and phylogenetic comparison on isolates was performed. For pathogenecity study of isolated PPMV-1, one hundred sixty day-old broiler chicks were divided into four equal groups. Groups 1 and 2 chicks vaccinated against ND by B1 vaccine at nine days. Groups 3 and 4 were kept as unvaccinated control groups. Groups 1 and 4 chicks were challenged with 10^5^EID_50 _of highest virulent isolated PPMV-1 by ocular route at day 29. The results indicated PPMV-1 is enzootic in Ahvaz pigeons and all isolates were virulent Newcastle disease virus with 112KRQKR*F117 motif. For study pathogenicity of pigeon isolate in chickens, they challenged with most virulent isolate, showed respiratory signs, conjunctivitis and in some cases depression and lethargy. In conclusion, isolated PPMV-1 is a virulent NDV and can infect chickens and produce mild ND in unvaccinated chickens.

## Introduction

Newcastle disease (ND) is a contagious viral disease in avian and affecting many species including chickens and pigeons and at least 239 species of other birds. This disease has a worldwide distribution and imposes a significant loss to the poultry industry all over the world. Virulent strain of avian paramyxovirus type 1 (APMV-1) is a causative agent of ND and known in pigeons as pigeon para-myxovirus type 1 (PPMV-1). The PPMV-1 is member of *Avulavirus* genus, *Paramyxoviridae* family and *Mono-negavirales* order. It is single-stranded, nonsegmented and negative-sense which enveloped RNA viruses with approximately 15 kb pairs genome.^1^ The PPMV-1 in pigeons has been identified and reported worldwide for a long time. There are many studies about detection and characterization of APMV-1 in pigeons.^1^^-^^5^ Bogoyavlenskiy *et al. *in Kazakhstan and Liu *et al.* in China isolated PPMV-1 in pigeons by using reverse transcription polymerase chain reaction (RT-PCR) with primers specific to the viral fusion protein (F) gene.^3^^-^^4^ Affected pigeons had clinical signs of paralyzed legs, wings or head tremors, torticollis, polydipsia, polyuria, anorexia, diarrhea and vomiting. There are many different studies on the pathogenesis of PPMV-1 in chickens. Some studies found isolated PPMV-1 responsible for panzootic Newcastle disease (ND) in chicken whereas others did not find it pathogenic for chicken.^1^^,^^6^^,^^7^^-^^13^ Infectivity of PPMV-1 to host cells is correlated with the cleavage glycoprotein fusion (F0) into F1 and F2 by host cells protease enzymes.^14^^,^^15^ The amino acids sequence of cleavage site determines proteases type for cleaving F0 and virulence of virus. In virulent isolates post-translation of F0 are cleaved by ubiquitous host proteases that found in all tissues. Base on the presence of a mono or multiple basic amino acid sequence motif at the F2 protein and a leucine at the F1 protein and a phenylalanine at the F1 protein, Newcastle disease virus (NDV) is divided into low virulent NDV or high virulent NDV, respectively.^16^^,^^17^ Phylogenetic analysis of F protein cleavage site in the APMV-1, isolates are divided into eleven genotypes and monoclonal antibody binding method classified it into six lineages. In most regions of the world, most recovered isolated genotype of PPMV-1, is VI genotype.^1^^,^^3^^,^^4^^,^^18^^,^^19^ Isolates of this genotype often cause nervous signs.^20^ Pchelkina *et al.* and Smietanka and Minta isolated PPMV-1 strains that possessed virulent F0 protein cleavage sites ^112^KRQKR*F^117 ^and these isolates were assigned to genotype VI.^19^^,^^21^ The PPMV-1 are characterized by phylo-genic analysis of genome sequences, intracerebral pathogenicity index (ICPI), intravenous pathogenicity index and mean death time (MDT) in chicken embryos, into typical lentogenic, mesogenic^3^^,^^12^ and velogenic NDVs.^12^^,^^22^ The PPMV-1 isolates increased their pathogenicity and virulence for chickens after one or more passage(s), and therefore, threat poultry production.^23^^,^^24^

Newcastle disease outbreaks with mortality in pigeons in the Ahvaz city of Iran have been observed. Due to lack of sufficient knowledge regarding PPMV-1 in Khuzestan province, the present work was designed for isolation and molecular identification of PPMV-1, comparing Ahvaz isolates with world isolates and evaluation of their pathogenicity in broiler chickens.

## Materials and Methods

**Virus isolation and identification. **Two hundred and thirty-two samples were collected from 50 unvaccinated pigeons lofts suspected to ND from private houses and bird markets from Ahvaz, Iran from November 2013 to May 2014. Cloacal and oropharyngeal swab samples of live pigeons (22 lofts) and samples from trachea, lung, liver, spleen, kidney, brain, proventriculus and cecal tonsil of pigeons suspected to ND were collected. Tissue and swab samples were transferred into Dulbecco's modified Eagle's medium (DMEM; Invitrogen, Carlsbad, USA) and then placed at –70 ˚C freezer until further processing. Isolation of the PPMV-1 was performed through intra-allantoic inoculation of 9- to 11- day-old embryonated chicken eggs. The allantoic fluid was collected and identified by standard hemagglutination (HA) and standard specific antisera to the reference strains of paramyxovirus and influenza virus. Harvested allantoic fluids were used as stocks and stored at –70 ˚C for propagation of one region of F gene that included cleavage site by polymerase chain reaction and next steps of study.^25^

**RNA extraction and RT-PCR. **Viral RNA was extracted from positive allantoic fluid using RNX^TM^-Plus Kit (CinnaGen, Tehran, Iran) according to the manufacturer’s instructions. At the final steps the extracted RNA was transferred to 30 μL diethylpryocarbonate water and stored at –70 ˚C. The cDNA synthesis was performed by using the daRT RT, cDNA synthesis kit (EURx Ltd., Gdansk, Poland) according to the manufacturer’s instructions using F gene primers that used in PCR. The RT-PCR was performed by the method described previously by Liu*et al*. Briefly, Two primers as 5'-ATG GGC (C/T)CC AGAC(C/T)CT TCT AC-3' (sense) and 5'-CTG CCA CTG CTA GTT GTG ATA ATC C-3' (antisense, in 582 nucleotides of the fusion protein [F] gene), were ordered for the synthesis to CinnaGen and used in RT-PCR. These two primers generated a 535 bp fragment including nucleotides 47 and 581 of the F gene.^4^ The PCR materials were provided from CinnaGen Co. and reactions were performed in a thermocycler (Quanta Biotech, London, UK) at 50 μL consisted of 10X PCR buffer, 1.50 μL of MgCl_2_ (50 mM), 10 μL of dNTP (10 mM), 0.50 unit of Taq-polymerase enzyme, 1 μL of two primers (10 pM), 5 μL of template DNA and 35 μL of RNase free water. The program for thermal cycling was 35 cycles of 94 ˚C for 1 min, 56 ˚C for 2 min, 74 ˚C for 1 min and finally 74 ˚C for 10 min. Then, PCR products were analyzed by electrophoresis on a 1.5% agarose gel.^4^

Sequencing of PCR products, analysis of nucleotide, deduced amino acid sequences and phylogenetic analysis. Twelve PCR products were sent for sequencing. After purification, the nucleotide sequencing was performed by using an automatic sequencer with bilinear reading, comfort read method (Bioneer, Daejeon, South Korea). Editing nucleotide sequence, analyzing, translating of amino acid sequences and alignments were performed with the CLC sequence viewer and CLC bio (Qiagen Co., Tehran, Iran). A fragment consisted of 369 nucleotides of the F gene, starting from TAG and including the F0 cleavage site was compared with the available sequences in GenBank. Finally, a phylogenic tree was generated by the neighbor-joining method.

**Pathogenicity and serological study. **Virulence of the isolates was determined by MDT in embryos according to the methods described by Hanson.^26^ The most virulent isolate was selected for phathobiology study in chickens. The PPMV-1/pigeon/Iran-14B (PPMV1-IR-14B) due to the highest MDT value among other eleven isolates of PPMV-1 (MDT = 62.40) was selected to challenge chickens. Embryonic infectious dose 50% (EID_50_) of isolate was calculated with Reed and Muench method in embryonated chicken eggs.^27^ One hundred and sixty one-day-old broiler chicks procured and after bleeding for determining of maternal antibody and vaccination time, were randomly divided into 4 equal groups. Group 1 and 2 chicks were vaccinated against ND by B1 vaccine at day 9 by oral route. Groups 3 and 4 chicks were kept as unvaccinated control groups. Chicks of all groups at day 28 were bled via wing web for determining antibody titer against NDV. Group 1 and 4 chicks at day 29 were transferred into new rooms and challenged with 10^5 ^EID50 isolated pigeon NDV by ocular route (0.10 mL per bird) and birds were observed three times a day up to 14 days. Presence of antibodies titer against NDV was measured by hemagglutination inhibition (HI) test at days 0, 7 and 14 post infection (DPI).^25^

## Results

**Fig. 1:**
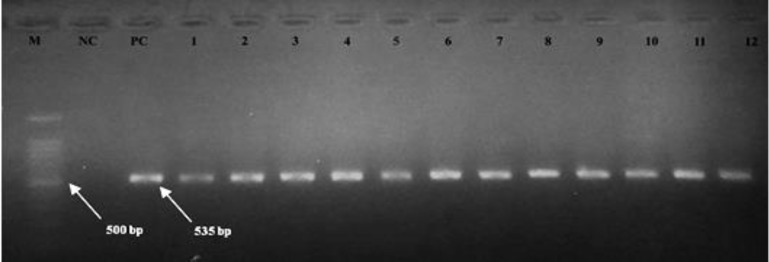
RT-PCR results of 12 positive PMV-1 samples performed by electrophoresis. Lane M: 100 base pair marker. Lanes 1-12: positive samples with 535 bp bands (535 bp bands indicate PMV-1 detection), Lanes PC and NC: positive control and negative control, respectively

**Virus isolation and identification. **Twelve virus isolates in embryonated eggs were positive by HA tests and confirmed by specified RT-PCR for PMV-1 (Fig. 1).

**Estimation of NDV isolates pathotype. **The MDT, molecular characteristics and accession numbers of PPMV-1 isolates recovered from pigeons in Iran during 2013-2014 are presented in Table 1. Pathogenicity of all isolates based on their MDT was characterized as mesogenic (ranging from 60 to 90 hr) with a range from 62.40 to 79.20 hr. Molecular pathotyping based on analyzing of deduced amino acid sequence of the cleavage site of F protein of all isolates was determined to be ^112^KRQKR*F^117 ^and found typical virulent NDV strains.^1^^,^^25^

**Fig. 2:**
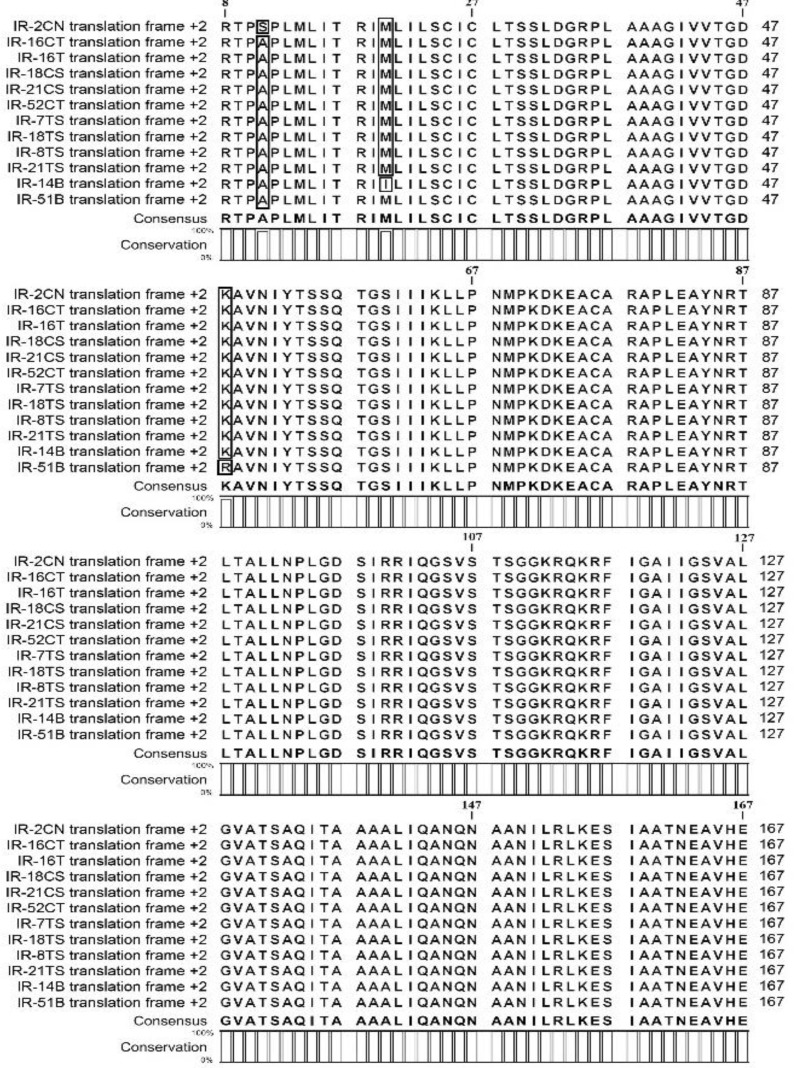
Aligned NDV amino acids sequences of the cleavage site of F protein of twelve PPMV-1 isolates. All sequences were truncated to 160 amino acids placing between amino acid positions 8 and 167

**Fig. 3:**
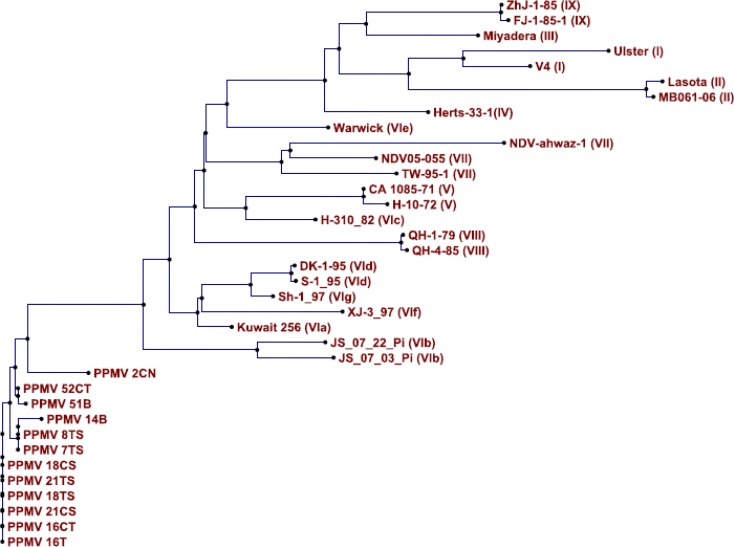
Phylogenic tree generated by the neighbor-joining method from twelve RT-PCR products amplified by the primer of this study in comparison with F gene sequences of selected NDV isolates registered in the GenBank. All NDV isolates are related to pigeon and are PPMV-1 and belonged to the genotype VI

**Amino acids sequences, genotyping and phylo-genetic relationships among PPMV-1 isolates. **Amino acids sequences of all twelve isolated PPMV-1 are displayed in Figure 2. Based on phylogenic evaluation, all pigeon isolates were belonged to genotype VI. Phylogenetic analysis of PPMV-1 strains isolated in Iran and relation-ships with other worldwide PPMV-1 isolates based on sequence analysis of the variable region of the F gene are shown in Figure 3.

**Pathogenicity and serological study. **All chickens challenged with PPMV1-IR-14B isolate (accession number: LC055507) were alive and showed respiratory symptoms (Table 2). The highest respiratory symptoms were observed in group 4 (vaccine + and challenge +) with 67.5% and average onset of symptoms was 1.70 DPI, mild periocular edema and conjunctivitis 25.00% with an average onset of symptoms 2.00 DPI, depression and lethargy 20.00% with onset of symptoms 4.20 DPI. In group 1, chicks received NDV B1 vaccine and challenged with PPMV1-IR-14B, 5.00% of chickens showed ND symptoms included 5.00% mild respiratory symptoms and 5.00% conjunctivitis. Groups 2 and 3 chicks did not show symptoms. 

**Table 1:** Mean death time, molecular characteristics and accession No. of PPMV-1 isolated from pigeons in Ahvaz, Iran during 2013-2014.

**Isolate identification**	**Abbreviation**	**Mean death time**	**Molecular pathotyping**	**Accession numbers**
**PPMV-1/pigeon/Iran-2CN**	PPMV1-IR-2CN	74.40	^112^KRQKR*F^117^	LC055504
**PPMV-1/pigeon/Iran-7TS**	PPMV1-IR-7TS	69.60	^112^KRQKR*F^117^	LC055505
**PPMV-1/pigeon/Iran-8TS**	PPMV1-IR-8TS	72.00	^112^KRQKR*F^117^	LC055506
**PPMV-1/pigeon/Iran-14B**	PPMV1-IR-14B	62.40	^112^KRQKR*F^117^	LC055507
**PPMV-1/pigeon/Iran-16T**	PPMV1-IR-16T	72.00	^112^KRQKR*F^117^	LC055508
**PPMV-1/pigeon/Iran-16CT**	PPMV1-IR-16CT	69.60	^112^KRQKR*F^117^	LC055509
**PPMV-1/pigeon/Iran-18TS**	PPMV1-IR-18TS	76.80	^112^KRQKR*F^117^	LC055510
**PPMV-1/pigeon/Iran-18CS**	PPMV1-IR-18CS	79.20	^112^KRQKR*F^117^	LC055511
**PPMV-1/pigeon/Iran-21TS**	PPMV1-IR-21TS	62.40	^112^KRQKR*F^117^	LC055512
**PPMV-1/pigeon/Iran-21CS**	PPMV1-IR-21CS	67.20	^112^KRQKR*F^117^	LC055513
**PPMV-1/pigeon/Iran-51B**	PPMV1-IR-51B	69.60	^112^KRQKR*F^117^	LC055514
**PPMV-1/pigeon/Iran-52CT**	PPMV1-IR-52CT	74.40	^112^KRQKR*F^117^	LC055515

**Table 2:** Clinical signs of isolated PPMV1-IR-14B inoculated in 29-day-old chickens

**Groups**	**Vaccine**	**Challenge**	**Any signs **	**Respiratory system**	**Nervous system **	**Digestive system **	**Conjunctivitis **	**Depression**	**Death**
**1**	+	+	5.00^a^	5.00a* (2)**	0.00	0.00	5a (3)**	0.00	0.00
**2**	+	-	0.00	0.00	0.00	0.00	0.00	0.00	0.00
**3**	-	-	0.00	0.00	0.00	0.00	0.00	0.00	0.00
**4**	-	+	67.50^b^	67.50b (1.70)**	0.00	0.00	25.00b (2.00)**	20.00b (4.20) **	0.00

* : frequency of birds, and

** : days, average onset of symptoms.

ab Different superscripts indicate significant differences within each column (*p *< 0.05).

Mean antibody titers against NDV in sera of groups 1, 2 and 4 chicks increased and the highest HI mean antibody titer against NDV was in group 1 chicks. Mean antibody titers against NDV in sera of group 3 did not increase (Table 3).

**Table 3:** Mean antibody titers in chickens before and after inoculation with PPMV1-IR-14B in 29-day-old chickens (HI mean [log_2_] ± STD

**Groups**	**Vaccine (B1, 9-day-old)**	**Challenge**	**-1 day**	**7 days**	**14 days**
**1**	+	+	4.70 ± 1.00 aA	3.20 ± 1.4 acA	7.00 ± 0.50 aB
**2**	+	-	4.70 ± 1.00 aA	5.30 ± 1.50 aA	5.20 ± 0.50 acA
**3**	-	-	0.50 ± 0.50 bA	0.40 ± 0.50 bA	0.20 ± 0.50 bcA
**4**	-	+	0.50 ± 0.50 bA	1.00 ± 0.50 bcA	6.90 ± 1.00 a B

abc Different lowercase superscripts indicate significant differences within each column (*p *< 0.05).

AB Different uppercase superscripts indicate significant differences within each row (*p *< 0.05).

## Discussion

Outbreaks of ND in pigeon have frequently occurred in Ahvaz, Iran. The disease remains a constant threat to commercial poultry and leads to huge economic losses. Furthermore, no information was available on NDVs in Ahvaz. Therefore, present work was designed for isolation and molecular identification of PPMV-1, comparing local isolates with the world isolates and evaluation of isolated PPMV-1 pathogenicity in broiler chickens. 

There are many studies about detection and characterization of APMV-1 in pigeons by RT-PCR.^3^^,^^4^^,^^24^ Primers used in the present study for detection and amplification of partial F gene were applied for all NDVs including PPMV-1 and were not specific for detection of PPMV-1.^4^

The PPMV-1 was classified in APMV-1 and for identification of PPMV-1, monoclonal antibody and recently phylogenetic comparison of partial or full sequence of fusion gene are applied.

Nowadays, phylogenetic analysis of genome sequences becomes a standard method in laboratories.^1^

Pathotyping of NDV with conventional methods such as MDT and ICPI is difficult and time-consuming, but new methods such as PCR and sequence analysis of F gene encoding the fusion protein cleavage site are very rapid and can differentiate virulent and avirulent isolates from each other.^10^^,^^16^^,^^28^ The deduced amino acid sequence of F protein cleavage site for all isolates was determined to be ^112^KRQKR*F^117 ^and isolates are virulent base on molecular characterization and OIE definition.^25^

Bogoyavlenskiy *et al.* in Kazakhstan isolated PPMV-1 in pigeon using RT-PCR with primers specific to the viral F protein gene. Cleavage site of viral F gene indicated motif ^112^KRQKR*F^117^ that was similar to motif of present study.^3^ Also, Pchelkina *et al*. isolated PPMV-1 strain that possessed virulent F0 protein cleavage sites ^112^KRQKR*F^117^and based on partial genome sequencing and phylogenetic analysis, the isolates were assigned to genotype VIb.^21^ In Poland, Smietanka and Minta isolated PPMV-1 from ornamental pigeons that showed the amino acid sequence at the cleavage site of F2/F1.^19^ Liu *et al*. in China characterized isolates recovered from pigeon with motif ^112^KRQKR*F^117 ^which are similar to our study and all isolates are belonged to VI Genotype.^4^ Mentioned studies are only studies in which isolated PPMV-1 from pigeons and these motives (^112^KRQKR*F^117^) have been observed.

In Africa, Asia and Europe most recovered isolated genotype of PPMV-1, is VI genotype.^3^^,^^4^^,^^18^^,^^19^^,^^24^ Isolates that belong to genotype VI often cause nervous signs.^20^ Twelve isolates in present study were belonged to genotype VI and the greatest similarity was observed with isolates from Kuwait (accession number: AF001109), Sweden (accession number: AF001131), Denmark (accession number: AF001129) and china (accession number: AF458018, FJ766526, FJ766531). According to amino acids sequences of 12 isolates, the isolated viruses were divided into four groups (Fig. 2).

Based on MDT, PPMV-1 isolates were ranged from 62.40 to 79.20 hr and characterized as mesogenic, many studies on MDT of PPMV-1 isolates have been performed in the world and mostly reported PPMV-1 isolates as mesogenic^3^^,^^12^^,^^24^ and some study reported as lentogenic group.^12^^,^^22^

Based on phylogenetic studies, it was concluded that PPMV-1 strains originally are transmitted from chicken to pigeon at first. Then, after a period of time, PPMV-1 isolates appear to have more virulence and adaptation for pigeons and lose their virulence and adaptation for chickens gradually.^1^ Outbreak of PPMV-1 in chicken is possible as it has occurred in the past.^2^^,^^9^

In this study, challenging chicken with PPMV1-IR-14B caused signs of disease in 67.50% of chickens in group 4 (vaccine – and challenge +) that this was significantly more than other groups. Challenging this isolate did not cause any nervous symptoms in challenged groups and the most prominent symptoms observed were respiratory (in 67.50% of chickens) and then conjunctivitis (in 25.00% of chickens). Depression and lethargy were seen in group 4 (vaccine - and challenge +). All symptoms were significantly lesser in group 1 (vaccine + and challenge +) compared with group 4 (vaccine - and challenge +) which showed group 1 (received B1 vaccine) has less morbidity than group 4 (vaccine - and challenge +). None of the birds in groups died indicating PPMV1-IR-14B used for challenging in this study could not cause death in chickens. 

Toro *et al.* showed that PPMV-1 with other infections could cause respiratory symptoms which were characterized by sneezing and restricted to upper respiratory tract.^13^ Kommers *et al*. by using RT-PCR, isolated 6 PPMV-1 and passage them to embryonated chicken eggs, then inoculated to chickens they found 10.00 to 20.00% mild periocular edema and bilateral conjunctivitis, two birds had slight depression, other isolates shown symptoms of tremors, drowsiness and incoordination but respiratory symptoms were not seen. In present study, some signs such as periocular edema, bilateral conjunctivitis and depression were seen.^11^ Pienaar* et al.* with inoculation of isolated PPMV-1 by ocular route in 0.10 mL of 10^7.5^ EID_50_ observed mild respiratory symptoms in 10.00% of birds and the results of which were similar to this study.^12^

The PPMV1-IR-14B in this study could induce humeral immune response in chickens. This virus could significantly increase antibody titer of chicken serum against standard antigen of NDV (Lasota) in HI test in group 4 (vaccine - and challenge +) rather than group 3 (vaccine - and challenge -), (*p *< 0.05). This increase was from 0.50 HI units (average) in day 28 to 6.90 in day 42 (13 days post challenge). Pienaar *et al.* found increase in HI titer against NDV after challenging four weeks chickens from 0.10 HI units in -1 DPI to 6.50 in 16 DPI. 

In conclusion, this study indicated that PPMV-1 is enzootic in pigeons and RT-PCR with general primers for APMV-1 could use to multiply F gene encoding the fusion protein cleavage site. Analysis of the 12 isolates F0 cleavage site showed motif ^112^KRQKR*F^117^ that is an indication of an acute NDV. Twelve isolates from this study were belonged to genotype VI and the most relationship was found between these isolates and isolates from Kuwait, Sweden, Denmark and china. Based on the phylogenetic analysis, 12 virus isolates were divided into 4 groups and all isolates were mesogenic and the chickens challenged with most virulent virus (PPMV1-IR-14B) only showed mild respiratory symptoms such as conjunctivitis and in some cases depression and lethargy. It was concluded that isolated PPMV-1 was a virulent ND based on the molecular characterization of cleavage site and could cause mild ND in chickens, whereas B1 vaccine could protect chickens against its morbidity and signs.
